# Regression of Intracranial Meningiomas Following Treatment with Cabozantinib

**DOI:** 10.3390/curroncol28020145

**Published:** 2021-04-18

**Authors:** Rupesh Kotecha, Raees Tonse, Haley Appel, Yazmin Odia, Ritesh R. Kotecha, Guilherme Rabinowits, Minesh P. Mehta

**Affiliations:** 1 Department of Radiation Oncology, Miami Cancer Institute, Baptist Health South Florida, Miami, FL 33176, USA; mohammed.tonse@baptisthealth.net (R.T.); HaleyA@baptisthealth.net (H.A.); MineshM@baptisthealth.net (M.P.M.); 2 Herbert Wertheim College of Medicine, Florida International University, Miami, FL 33199, USA; YazminO@baptisthealth.net (Y.O.); GuilhermeR@baptisthealth.net (G.R.); 3 Department of Neuro Oncology, Miami Cancer Institute, Baptist Health South Florida, Miami, FL 33176, USA; 4 Department of Medicine, Memorial Sloan Kettering Cancer Center, New York, NY 10065, USA; kotechar@mskcc.org; 5 Department of Medical Oncology, Miami Cancer Institute, Baptist Health South Florida, Miami, FL 33176, USA

**Keywords:** meningioma, cabozantinib, VEGF, targeted therapy

## Abstract

Recurrent meningiomas remain a substantial treatment challenge given the lack of effective therapeutic options aside from surgery and radiation therapy, which yield limited results in the retreatment situation. Systemic therapies have little effect, and responses are rare; the search for effective systemic therapeutics remains elusive. In this case report, we provide data regarding significant responses in two radiographically diagnosed intracranial meningiomas in a patient with concurrent thyroid carcinoma treated with cabozantinib, an oral multitarget tyrosine kinase inhibitor with potent activity against MET and VEGF receptor 2. Given the clinical experience supporting the role of VEGF agents as experimental therapeutics in meningioma and the current understanding of the biological pathways underlying meningioma growth, this may represent a new oral therapeutic alternative, warranting prospective evaluation.

## 1. Introduction

Meningiomas account for approximately one-third of primary central nervous system tumors in adults. Although most meningiomas are well-differentiated, with low proliferative capacity [1], recent analyses using the updated grading criteria adopted by the World Health Organization (WHO) have demonstrated that as many as 15% to 20% should be classified as atypical WHO Grade II [2]. In the Radiation Therapy Oncology Group (RTOG) 0539, a phase II cooperative group trial assessing the safety and efficacy of risk-adapted meningioma treatment strategies, “high-risk” meningioma patients (WHO grade II subtotal resection, recurrent grade II, or any grade III), experienced a 3-year progression-free survival (PFS) of only 59%, with a 3-year local control rate of only 69% [3], prompting broad agreement for the need for more effective multimodality therapy, and the optimization of treatment strategies in this patient population.

Evaluation of systemic therapies in recurrent and higher grade meningiomas suggest that most chemotherapeutic agents have minimal to no activity against this disease group. Agents such as hydroxyurea, dacarbazine, ifosfamide, temozolomide, irinotecan, and α-interferon have demonstrated no clinical efficacy [4]. The pathogenesis of meningioma is incompletely understood, and studies have now focused on gene expression profiling and DNA methylation arrays to characterize transcriptional and epigenetic changes in the hope of identifying prognostic markers and molecular drivers. Emerging data have identified the upregulation of pathways involved in growth, proliferation, and angiogenesis, including EGFR, mTOR, PDGF, and VEGF, suggesting these as potential actionable targets [5]. Moreover, high MET expression has been demonstrated to be a predictor for recurrence in meningioma [6].

Markers of angiogenesis, including increased vessel density and VEGF expression, are detectable in meningiomas, and have been linked with grade and prognosis [7,8]. At least two VEGF targeting agents have previously been tested in this setting: bevacizumab and sunitinib. Bevacizumab showed evidence of antitumor activity, including radiographic responses and prolonged PFS in a retrospective series [9]. A prospective, multi-center single-arm phase II trial investigated the efficacy of sunitinib, a small molecule tyrosine kinase inhibitor (TKI), against VEGFR and PDGFR in 36 patients with WHO grade II and III recurrent or progressive meningiomas, resulting in a very modest median PFS of 5.2 months [10]. Reports focused on recurrent WHO grade I meningiomas are limited. A phase II study evaluated the combination of bevacizumab and everolimus, an mTOR inhibitor, in patients with recurrent or progressive meningioma (*n* = 5 WHO grade I, 17 total) [11]. Interestingly, the median PFS was greater for WHO grade II and III tumors (22 months) as compared to grade I tumors (17 months). Similarly, another phase II study investigated the efficacy of Vatalanib, a small molecule protein kinase inhibitor with activity against VEGF receptors, PDGFR-beta, and c-kit in refractory meningiomas (*n* = 2 WHO grade I, 25 total), with overall modest activity [12]. Together, these studies demonstrate the promise of VEGF inhibitors in meningioma.

Cabozantinib is a multitarget oral TKI with potent activity against MET and VEGF receptor 2 (VEGFR2) [13]. It is FDA-approved for the treatment of progressive metastatic medullary thyroid cancer and advanced renal cell carcinoma. In this case study, we report a patient with metastatic thyroid cancer treated with cabozantinib who demonstrated a marked response in two previously untreated, incidental intracranial meningiomas. To the best of our knowledge and based on a search of the medical literature (MEDLINE, accessed on 29 February 2020), this represents the first documented case of a response to this oral agent in meningioma and provides an encouraging example for further study.

## 2. Case Report

A 65-year-old male with a past medical history significant for medically controlled hypertension presented initially with dysphonia and was found to have a large thyroid mass displacing the trachea and extending into the superior mediastinum. He underwent a total thyroidectomy; pathology revealed follicular thyroid carcinoma, insular variant, and he was treated with 200 mCi of oral radioactive iodine (RAI). A seven-day post-iodine scan revealed evidence of significant residual iodine-avid tissue within the thyroid bed, as well as multiple non-calcified pulmonary nodules with increased iodine uptake suspicious for bilateral metastatic pulmonary disease. He later developed symptomatic local recurrence, as well as an increase in the burden of distant metastatic disease, approximately 1 year after resection, and 9 months after RAI. He was treated with Lenvatinib 20 mg PO once daily. After a few weeks, he continued to have persistent symptoms of vocal cord paralysis and, given concern for persistent disease, underwent total laryngopharyngectomy, cervical esophagectomy, and post-operative external beam radiotherapy, and his systemic therapy was discontinued. MRIs of the spine at that time revealed a T12 metastasis with an epidural tumor treated with stereotactic body radiotherapy.

**Figure 1:**
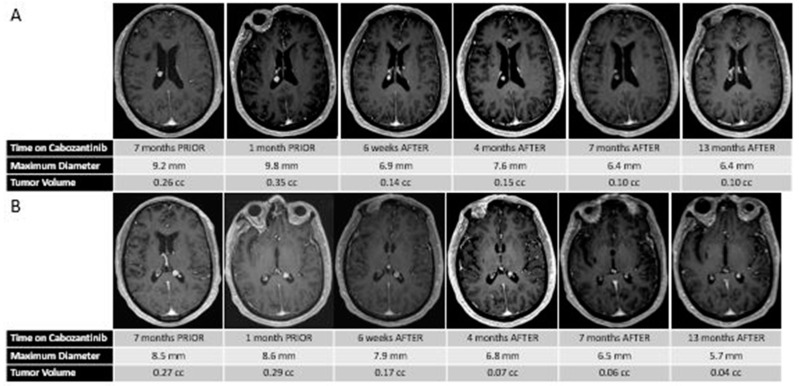
(**A**) Sequential MR images demonstrating the changes in size and volume of the right putative intraventricular meningioma before and after treatment with cabozantinib. (**B**) Sequential MR images demonstrating the changes in size and volume of the left putative intraventricular meningioma before and after treatment with cabozantinib.

At age 70, five years after initial presentation, he presented with headaches, and an MRI scan of the brain revealed a left posterior frontal parafalcine lesion measuring 2 × 1.5 × 2.5 cm, along with two stable intraventricular meningiomas (measuring approximately 8.5 and 9.2 mm each). Interval brain MRI performed 2.5 months later, at the time of transfer to our center, revealed that the left parafalcine mass had increased in size to 3.5 × 3.4 × 2.4 cm, with moderate mass effect on the motor strip, with the two intraventricular putative meningiomas stable in size. Given the patient’s progressive weakness and lesion size, he underwent resection of the left posterior frontal parafalcine mass, and pathology confirmed metastatic thyroid carcinoma consistent with his known primary. He underwent post-operative stereotactic radiosurgery to the surgical cavity and continued observation was recommended for the putative stable meningiomas. Subsequent restaging studies revealed progressive metastatic cancer, with an increase in the size and number of metastatic pulmonary nodules, nodal progression in the neck, and enlarging osseous metastases. His two-month post-treatment brain MRI revealed no evidence of progression of the disease within the surgical cavity and the continued stability of the two intraventricular meningiomas. He was started on systemic therapy with cabozantinib 60 mg PO daily for his metastatic thyroid cancer, which was dose-reduced 6 weeks later to 40 mg PO daily due to modest fatigue. Interval follow-up brain MRI performed 8 weeks following treatment of the brain metastasis and 6 weeks post-cabozantinib demonstrated no radiographic recurrence at the surgical bed, but an unexpected and dramatic reduction in size and volume of the intracranial meningiomas (60% and 40% volumetric reduction) (see Figure 1). This response was sustained at an interval brain MRI performed for the surveillance of brain metastasis approximately 3 months on cabozantinib and continued until his most recent follow-up MRI performed approximately 1 year after starting therapy.

## 3. Discussion

Herein, we describe a significant and unexpected early intracranial response to two intracranial meningiomas in a patient treated with cabozantinib. This response remains in line with prospective evidence demonstrating activity of VEGF directed therapies in meningiomas, but represents the first report, to our knowledge, using the potent VEGFR TKI cabozantinib. Given the current understanding of the biological mechanisms underlying meningioma growth, the tolerability of the agent, and the ease of oral administration, this case example provides rationale for prospective investigation of disease-specific activity.

Several studies and reports have detailed the activity of VEGF directed therapies for patients with meningiomas, especially for those with high-grade/recurrent disease. Due to the differences in doses used across the studies, as well as the duration of treatment, heterogeneity of patient selection, variation in prior radiotherapy, the potential for diagnostic uncertainty in radiation necrosis vs. true tumor progression, and the lack of long-term prospective follow-up, the data remain inconclusive (See Table 1).

Sunitinib demonstrated promising activity in a phase II meningioma trial [10]; however, in untreated renal cell carcinoma brain metastasis, the objective response rate was 0% [23]. There are currently only three ongoing phase II prospective trials for patients with sporadic recurrent or progressive meningioma: one using bevacizumab monotherapy (NCT01125046), another studying a combination of bevacizumab and electric field therapy (NCT02847559), and a third evaluating the activity of a single agent PD-1 inhibitor Pembrolizumab (NCT03279692) (see Supplemental Table S1).

Cabozantinib is a small molecule inhibitor of multiple tyrosine kinases including MET, VEGFR2, RET, and AXL. Selective inhibition of VEGFR2 may lead to increased invasiveness and metastasis, and preclinical models suggest that the inhibition of VEGFR2, together with c-MET, may decrease tumor size and invasiveness [24]. Cabozantinib was FDA approved in 2012 for patients with progressive metastatic medullary thyroid cancer based on improved PFS compared to placebo (11.2 months vs. 4.0 months, *p* < 0.001) [25]. In 2016, cabozantinib was approved for adults with advanced renal cell carcinoma following prior antiangiogenic therapy and, subsequently, in the first-line setting [26]. There has also been documented experience of treatment of primary intracranial tumors with cabozantinib. A phase II trial examined the efficacy and safety of cabozantinib in 222 patients with progressive or recurrent GBM, and initial results demonstrated modest clinical activity in patients who had received prior antiangiogenic therapy [27]. More promising activity was observed in a subsequent subset of 152 patients not previously treated with antiangiogenic therapy (ORR of 15% for the combined dosing cohorts and a median PFS of 3.7 months in both cohorts) (see Supplemental Table S2) [28]. Therefore, this agent likely has intracranial penetration (not a requirement for meningioma) based on the limited efficacy in recurrent GBM.

Several limitations are to be noted from this case example. First, the lack of histopathological evaluation of the intraventricular lesions prevents a definitive diagnosis from being made and precludes biomarker evaluation. However, this was not clinically indicated in this patient, as the intraventricular lesions were present and stable during the patient’s cancer course and responded to the systemic therapy (unexpectedly); therefore, pathological evaluation has not been necessary. We do consider these lesions to be meningiomas versus brain metastasis for the following reasons: (1) the development of the single brain metastasis and its progression in size while treatment was arranged in the setting of the intraventricular lesions remaining stable during interval imaging; (2) the rarity of intraventricular brain metastases, representing only 1–5% of cases in general [29] and only a single case report of an intraventricular thyroid cancer metastasis reported in the literature [30]; (3) radiographic and biopsy-proven extracranial disease progression now over 1 year from the initiation of cabozantinib, along with intracranial disease progression with development of additional intracranial lesions, but continued response and stability of the intraventricular lesions, which would be strikingly discordant. An additional limitation to note is that this particular patient was presumed to have low-grade meningiomas based on their lack of growth trajectory and imaging appearance and responded to single agent Cabozantinib. Therefore, although similar VEGFR-directed agents have been utilized in those with higher grade recurrent or progressive disease, the responses may differ across different grades of meningioma, and the evaluation of the molecular underpinnings are key to better understanding the response assessments.

Additionally, the development of non-invasive or liquid biomarkers are equally important, especially if pathological evaluation is unable to be performed, such as in this particular case. Recently, non-invasive techniques, such as liquid biopsy or CSF analysis, have emerged as viable options in this space [31,32]. Circulating microRNAs (miRNAs) and DNA methylation hold great promise as novel clinical blood-based biomarkers for meningioma diagnosis and prognosis [33,34]. Currently, many more biomarkers are being identified, and research on the effectiveness of these techniques is being performed to determine their usefulness for the detection and monitoring of genomic alterations [31]. Additionally, we also considered, in this case, whether the radiographic response was primarily due to vasculature changes as a result of the targeted therapy or a direct effect on the meningioma cells, as it is known that meningiomas do not show an angiogenic switch involving VEGF, as is the case with gliomas. Nevertheless, the biological activity of VEGF and the molecular underpinnings behind meningioma proliferation suggest that it is a potential target for antiangiogenic therapy in meningiomas of all WHO grades.

## 4. Conclusions

Meningiomas are common tumors in a neuro-oncology practice, but there are few systemic options for patients with recurrent disease. Clinical experience with VEGF inhibitors demonstrates modest results. This case of a patient treated with the oral agent cabozantinib, with potent activity against MET and VEGFR2, may represent a new therapeutic option in this patient population with a highly unmet need. Unfortunately, we did not have actual tumor tissue from any of the lesions, as resection was not clinically indicated and therefore correlating the response with biomarker expression is not possible. Moreover, these lesions were stable over multiple MRIs, and radiographically most consistent with meningioma, unlike the known and resected thyroid metastatic lesion in the brain. Given the preclinical understanding of the molecular underpinnings of meningioma and the lack of effective systemic therapeutic options, a prospective evaluation of Cabozantinib is encouraged and warranted in patients with relapsed or refractory disease. This study is currently in development with the objectives of evaluating the PFS, overall response rate, overall survival, safety and tolerability, and quality of life for patients treated for recurrent meningioma of WHO Grades I–III. Correlation of MET expression, angiogenesis receptor status, and inflammatory signatures by gene expression profiling with response to cabozantinib on pre-treatment tissue samples will lead to a better understanding of this potential therapeutic modality. Similar initiatives are encouraged in this space to help provide alternatives for this challenging disease.

## Figures and Tables

**Table 1:** Selected clinical studies evaluating the use of VEGF agents in meningioma patients.

Author and Year	Studied Drug	Mechanism of Action	Study Type	Number of Patients	Prior Surgery	Prior RT *	WHO Grade Inclusion	PFS 6 **
Puchner et al., 2010 [14]	Bevacizumab	Anti-VEGF antibody	Case report	1	1	1	III	NA
Goutagny et al., 2011 [15]	Bevacizumab	Anti-VEGF antibody	Case report	1	NA	NA	NA	NA
Lou et al., 2012 [16]	Bevacizumab	Anti-VEGF antibody	Retrospective	14	14	11	I,II,III	86%
Nayak et al., 2012 [17]	Bevacizumab	Anti-VEGF antibody	Retrospective	15	15	15	II,III	44%
Nunes et al., 2013 [18]	Bevacizumab	Anti-VEGF antibody	Retrospective	15	NA	NA	NA	93%
Hawasli et al., 2013 [19]	Bevacizumab, Pazopanib	Anti-VEGF antibody, TKI	Retrospective	10	9	5	NA	NA
Raizer et al., 2014 [12]	Vatalanib	VEGFR + PDGFR TKI	Phase II	17	16	12	I,II,III	60%
Alanin et al., 2015 [20]	Bevacizumab	Anti-VEGF antibody	Retrospective	7	NA	NA	NA	NA
Kaley et al., 2015 [10]	Sunitinib	VEGFR + PDGFR TKI	Phase II	36	36	35	II,III	42%
Furtner et al., 2015 [21]	Bevacizumab	Anti-VEGF antibody	Retrospective	5	NA	NA	II,III	NA
Grimm et al., 2015 [22]	Bevacizumab	Anti-VEGF antibody	Phase II	40	40	40	I,II,III	27%
Shih et al., 2016 [11]	Bevacizumab, Everolimus	Anti-VEGF antibody	Phase II	17	16	12	I,II,III	69%

* RT = radiotherapy; ** 6-month progression-free survival.

## Data Availability

Not applicable.

## Supplementary Materials

The following are available online at https://www.mdpi.com/article/10.3390/curroncol28020145/s1. Table S1. Ongoing trials of VEGF agents in meningioma patients; Table S2. Select clinical studies evaluating Cabozantinib in patients with primary or metastatic CNS tumors.
