# Supplementary material for: Regression of Intracranial Meningiomas Following Treatment with Cabozantinib
Source: Curr Oncol. 2021 Apr 18;28(2):1537–43. doi: 10.3390/curroncol28020145 (PMC8167720; doi:10.3390/curroncol28020145)
Supplement: Supplementary file 1 [file curroncol-28-00145-s001.zip › curroncol-1176351-supplementary.pdf]

Supplementary Materials

Regression of Intracranial Meningiomas Following Treatment with Cabozantinib

Rupesh Kotecha, Raees Tonse, Haley Appel, Yazmin Odia, Ritesh R. Kotecha, Guilherme Rabinowits and Minesh P. Mehta

Table S1. Ongoing trials of VEGF agents in meningioma patients.

Trial Registration Number	Regimen	Mechanism of Action	Study Design	Number of Patients	WHO Grade Inclusion	Primary End Point*	Estimated Primary Completion Date
NCT01125046	Bevacizumab	Anti-VEGF antibody	Phase II	50	I/II/III	PFS - 6 Months	Jul-19
NCT02847559	Bevacizumab + Optune	Anti-VEGF antibody, Electric Field therapy	Phase II	27	II/III	PFS - 6 Months	Sep-21
NCT03279692	Pembrolizumab	PD-1 inhibitor	Phase II	26	II/III	PFS - 6 Months	Sep-25

* 6-month progression-free survival.

Table S2. – Select clinical studies evaluating Cabozantinib in patients with primary or metastatic CNS tumors.

Author and year	Regimen	Study Design	Number of Patients	Diagnosis
Wen et al., 2017 [17]	Cabozantinib	Phase II	152	Recurrent Glioblastoma
Cloughesy et al., 2018 [16]	Cabozantinib	Phase II	222	Recurrent Glioblastoma
